# *In vivo* detection of vertical root fractures in endodontically treated teeth: Accuracy of cone-beam computed tomography and assessment of potential predictor variables

**DOI:** 10.4317/jced.57471

**Published:** 2021-02-01

**Authors:** Marcela Quintero-Álvarez, Leslie-Melissa Bolaños-Alzate, Paula-Andrea Villa-Machado, Felipe-Augusto Restrepo-Restrepo, Sergio-Iván Tobón-Arroyave

**Affiliations:** 1 Senior Resident, Graduate Endodontic Program, Faculty of Dentistry, University of Antioquia. Medellín, Colombia; 2 Associate Professor. Graduate Endodontic Program, Faculty of Dentistry, University of Antioquia. Medellín, Colombia; 3 Titular Professor. Laboratory of Immunodetection and Bioanalysis, Faculty of Dentistry, University of Antioquia. Medellín, Colombia

## Abstract

**Background:**

This study aimed: (a) to determine the diagnostic performance of cone-beam computed tomography (CBCT) for detection of vertical root fractures (VRFs); (b) to evaluate the predictive value of diagnostic criteria regarding the definition of VRFs; and (c) to examine the robustness of the association of patient-, tooth-, and treatment-related variables with VRFs.

**Material and Methods:**

130 root-filled teeth with signs/symptoms of VRFs underwent clinical and CBCT assessments. Definite diagnosis of VRF was confirmed by endodontic microsurgical (EMS) exploration. Determination of diagnostic performance of CBCT was based on standard algorithms derived from two-way contingency table analysis. Predictive value of diagnostic criteria and the association between predictor variables with VRFs were analyzed using logistic regression models.

**Results:**

VRFs were detected during EMS in 50% of the teeth. Based on the finding of fracture lines on CBCT scans, sensitivity, specificity, and accuracy were 86.2%, 13.8%, and 50%, respectively. Teeth having more than three diagnostic criteria present had significant higher odds for VRF diagnosis. After logistic regression analysis, parafunctional habits, one-canal roots, excessive root canal enlargement, and absence of intra-radicular posts remained as robust predictor variables of VRFs.

**Conclusions:**

Although the sensitivity of CBCT for VRFs detection is high, the risk of false-positive results related to its low specificity makes that all suspected cases must be confirmed by surgical exploration. VRFs cannot be reliably diagnosed by isolated clinical signs/symptoms; instead those teeth possessing more than three diagnostic criteria might be considered practically pathognomonic. The parafunctional habits, one-canal roots, excessive root canal enlargement, and the absence of intra-radicular posts may act strongly/independently for the occurrence of VRFs in endodontically treated teeth.

** Key words:**Cone-beam computed tomography, diagnostic accuracy, diagnostic surgery, predictor variables, root canal treatment, vertical root fracture.

## Introduction

Despite of the high success rate of root canal treatment, endodontic failures may occur for multiple reasons, among which vertical root fractures (VRFs) play a crucial role. VRF is defined as the rupture of the tooth root structure that extends along the vertical axis of the roots (1,2). It has been well documented that they may occur during or after root canal treatment, although the occurrence in non-endodontically treated teeth has been also described (3). They may be complete, involving both sides of the root, and incomplete, involving one side (4-7).

It has been stated that VRFs constitute the third most common cause of tooth loss after dental caries and periodontal disease (8). Nevertheless, the data reported regarding their prevalence have been inconsistent, since clinical studies of endodontically treated teeth with suspected VRFs suggest that it may vary from 2% to 20%, as described in several reviews on this topic (9-11). This wide variation in the reported frequencies may be due not only to the methodological diversity of the studies (i.e., study design, sample size, case selection, diagnostic methods, and statistical analyses), but also to the ambiguous clinical presentation of VRFs (12).

When a VRF occurs, it extends to the periodontal ligament, thus inducing an inflammatory process in the adjacent periodontal tissue due to bacterial contamination from oral cavity (13,14), which in turn leads to the granulomatous tissue formation and periodontal breakdown (15). The detection of a VRF is challenging and its distinction usually requires a clinical and radiographic correlation, and sometimes the surgical exploration (5). Clinical signs and symptoms include localized periodontal swelling or abscess, sinus tract, tooth mobility, tenderness to palpation/percussion, and isolated periodontal pockets (16). Likewise, the foremost radiographic findings are represented by periradicular/lateral radiolucency, J-shaped radiolucency (bone loss halo), periapical radiolucency, and/or furcation involvement (12,16). Notwithstanding, at date, there is not substantial evidence in the literature that proves the accuracy of clinical and radiographic findings in terms of diagnosis and identification (15).

In addition to the former, the majority of the clinical studies assessing the aetiology of VRFs have been focused in the influence of individual risk variables on fracture generation after root canal treatment/retreatment, and actually few clinical studies have analyzed the combined effect of multiple variables (16-19). Nevertheless, the results obtained from these studies have been inconsistent, so that no firm conclusions can be established. Considering that overlap of adjacent structures in two-dimensional radiographs (i.e., conventional radiography) may limit the detection of fracture lines (5), and that cone-beam computed tomography (CBCT) can provide a more reliable method to analyze risk variables associated with the fracture generation, this study intended (a) to determine the diagnostic performance of CBCT for detection of VRFs; (b) to evaluate the predictive value of clinical and CBCT diagnostic criteria regarding the diagnosis of VRFs; and (c) to examine the robustness of the association of different patient-, tooth-, and treatment-related variables with development of VRFs.

## Material and Methods

-Study setting and design

This cross-sectional study was approved by the Institutional Ethics Committee for Human Studies of the University of Antioquia in Medellín, Colombia (Concept Number 35-2019) and carried out at the Faculty of Dentistry following the ethical guidelines of the Helsinki declaration. The sample size for preliminary clinical screening was calculated on the basis of patient population with diagnosis of persistent/emergent apical periodontitis (AP) referred for endodontic microsurgery (EMS) to the Postgraduate Endodontics Clinic between February 2017 and February 2020. Considering an original cohort of 195 referred patients and a case/control ratio = 1, the power calculation using a web-based statistical sample size calculator (Raosoft® Inc., Seattle, WA, USA) generated a sample size requirement of minimum 130 participants to reach a 95% confidence level, with an alpha value of 5% and a power >84% in identifying significant differences in the between-group comparisons. Eligibility criteria included patients with diagnosis of persistent or emergent symptomatic/asymptomatic AP after root canal treatment/retreatment and indication of EMS. Both teeth with and without periodontal local swelling, tenderness to percussion/palpation, discomfort on biting, increased mobility, sinus tracts, and/or isolated periodontal pocket formation were included. Conversely, exclusion criteria applied were: lack of information about root canal treatment procedure (i.e., date of completion, shaping technique and/or filling technique), absence of root canal filling, history of acute dento-alveolar trauma, evidence of visible fractures at clinical inspection, presence of caries or non-carious lesions on root surfaces, moderate-to-extensive coronal caries lesions, immature teeth endodontically treated, previous EMS, and severe generalized periodontitis.

Two trained observers (M. Q-A. and L.M. B-A.), calibrated regarding diagnostic criteria, examination procedures, and documentation formats by means of a joint assessment of written criteria, supplemented by drawings and clinical photographs, simultaneously examined all participants to avoid a biased interpretation of the data that might undermine the reliability of the results. When conflicting data were established between the observers, new assessments were accomplished and any further disagreement was resolved by discussion and consensus. Data pertaining to the history of root canal treatment such as type of treatment, evolution course, shaping technique, and filling technique, were obtained from the clinical records provided by those dentists who had performed the treatment. Also, the clinical records of patient- and tooth-related candidate predictor variables for association with VRFs included age, gender, parafunctional habits (bruxism or clenching evidenced by wear of the occlusal and incisal surfaces of the teeth and restorations), tooth type, tooth location, use as abutment, type of coronal restoration, and quality of coronal restoration. The conditions for a satisfactory coronal restoration included smooth transition of exploration probe across restoration margin, absence of marginal discrepancy, no clinical or radiographic signs of caries, and no history of crown decementation (20). In addition, the clinical signs and symptoms used for diagnosis of VRFs included the presence of isolated periodontal pockets ≥5 mm, sinus tracts, tenderness to percussion/palpation, increased tooth mobility, and periodontal swelling/abscess.

After clinical examination, CBCT images were obtained from each tooth using a 3D-Accuitomo 80® unit (J. Morita® Manufacturing Corp., Kyoto, Japan) operated at 80 kVp, 4-5 mA, 4 x 4 cm of field vision, voxel size 0.125 x 0.125 x 0.125 mm, 12 or 8-bits, and 17 seconds of exposure time. CBCT images were archived using One Volume Viewer Software (J. Morita® Manufacturing Corp.) for their sequential examination in the three orthogonal planes (axial, sagittal and coronal) by the same researchers that achieved the clinical data collection (M. Q-A and L.M. B-A), under ideal light conditions according to previously described guidelines (21) and using the magnification tool to enhance the images.

The evaluated diagnostic CBCT parameters included the record of presence/absence of hypodense lines and the periapical status. Before the survey, the examiners had been calibrated using printed instructions regarding CBCT image interpretation and software manipulation, along with reference images illustrating different periapical conditions and examples of fractured roots in CBCT scans. The detection of a hypodense line crossing the root completely or partially on at least two consecutive slices was the main radiographic feature for detecting a VRF (22,23). Periapical status was recorded for each teeth following previous defined criteria (14) as: normal periapical structures (no periapical rarefaction detected); periradicular/lateral hypodensity (rarefaction limited to the lateral aspect of the affected root, without involving the coronal or apical regions); J-shaped hypodensity (periradicular rarefaction observed on the lateral aspect of the affected root, which extended apically and to the opposite side of the root); periapical hypodensity (rarefaction located in the periapical region of the affected tooth); and furcation involvement (rarefaction observed in the furcation area only). Otherwise, data concerning tooth morphology, involved root, number of canals of the affected root, current crown-to-root ratio, root curvature angle, root canal enlargement, presence of intra-radicular posts, as well as the apical extension and density of root canal filling were all recorded as CBCT predictor variables and included either within tooth- or treatment-related predictor variables when appropriate. Root canal enlargement was categorized arbitrarily into two subgroups based on the root thickness as observed on CBCT images (i.e., adequate, ≤one-third of root width vs. excessive, beyond one-third of root width). The conditions for an adequate root canal filling included absence of voids, adaptation to the lateral canal walls (homogeneity), and length from 0-2 mm short of the radiographic apex (24).

Subsequently, definite diagnosis of VRF was confirmed by surgical exploration. The informed consent of all patients was obtained after the nature of the procedure and possible discomforts and risks were fully explained. After acceptance, microsurgical procedure was achieved under infiltrative and regional anesthesia by two experienced endodontists and researchers (P.A. V-M. and F.A. R-R.) blinded to the initial records, to ascertain objectivity and consistency, and using an operating microscope (OPMI Pico, Carl Zeiss®, Oberkochen, Germany). After the elevation of full-thickness flaps, the granulomatous tissue present on the roots was removed and root surface was stained with 1% methylene blue solution to be inspected under X10 to X25 magnifications for the presence of fracture lines. When no VRFs were detected, EMS approach was completed as previously described (25). On the contrary, when a fracture line was noticed, a decision was made to either perform a root resection or tooth extraction plus guided tissue regeneration when indicated. Based on definitive microsurgical findings two clinical groups were established for comparisons (i.e., Non-VRF group vs. VRF group).

-Statistical methods and data analysis

For statistical processing of data, several steps were required. Initially, the diagnostic performance of CBCT for determination of VRFs was performed using an online calculator (http://StatPages.info/ctab2x2.html). Afterward, data analysis was performed using the SPSS® 25.0 statistical program (IBM, Armonk, NY). Bivariate comparisons were achieved using Pearson’s chi-square (χ2) test or Fisher exact test, when indicated, to detect differences in relation to the clinical/CBCT parameters that would allow to build a predictive model for diagnosis of VRFs, and to establish differences in patient-, tooth-, and treatment-related variables that could act as potential predictor variables for association with VRFs. The predictive model for diagnosis of VRFs was constructed using a logistic regression model based on the number of positive clinical and CBCT diagnostic criteria present adjusting for demographic characteristics. In addition, those variables significantly associated with VRFs in the bivariate comparisons were analyzed by univariate and multivariate binary logistic regression analyses adjusting for non-significant confounding covariables with P-value ≤0.20 to determine the strength and independence of the associations. Positive associations were considered valid when the odds ratio (OR) was greater than 2 and the confidence interval (CI) was >1.0. P-values <0.05 were regarded as statistically significant. The Hosmer-Lemeshow and the c-statistic tests were used to evaluate the calibration and discrimination power of the multivariate models, respectively.

## Results

-Diagnostic performance of CBCT for determination of VRFs

The analysis of the diagnostic performance of CBCT showed a sensitivity (i.e., proportion of true positives that are correctly identified by the test) relatively high (86.2%) and a specificity (i.e., the proportion of true negatives that are correctly identified) very low (13.8%) for detecting VRFs. In addition, positive predictive value (PPV), i.e., the proportion of teeth with VRFs that were correctly diagnosed, was 50%, and negative predictive value (NPV), i.e., the proportion of teeth with no fractures that were correctly diagnosed, was 50%, so that the overall accuracy was only 50%.

-Clinical and CBCT profile of the study population

**Figure 1:**
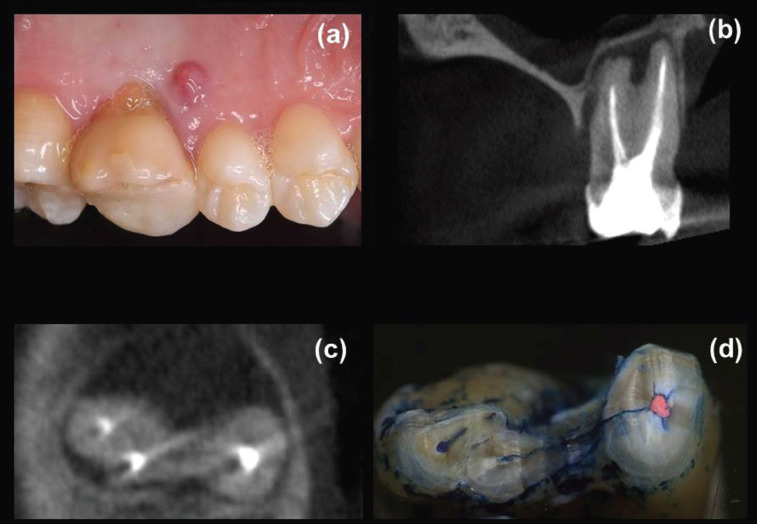
Maxillary left first molar with symptomatic apical periodontitis and concomitant marginal lesion with communication. (a) Clinical presentation of the tooth and its surrounding palatal mucosa before the surgical exploration. A sinus tract opening located near gingival margin is observed. (b) A coronal CBCT image showed a vertical palatal alveolar bone loss reaching the apical region thereby producing a J-shaped radiolucency (bone loss halo). (c) Magnified axial CBCT view revealed a bucco-lingually oriented hypodense line (arrows) extending from the disto-buccal to the palatal roots and extensive periradicular bone loss in the mesial aspect of the tooth. (d) After root-end resection and methylene blue staining, the extracted tooth showed the fracture line running through the furcation area.

**Figure 2:**
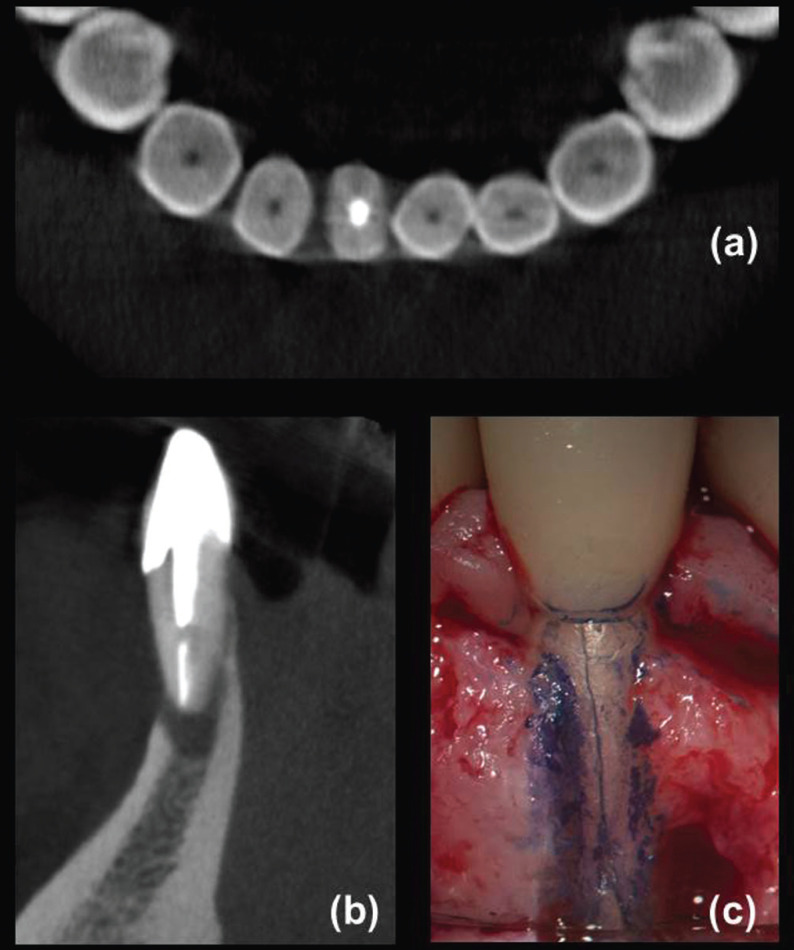
Surgical finding of a fracture line in a mandibular right central incisor not detected on the CBCT scans. (a) Magnified axial CBCT scan showing absence of hypodense lines and any other radiological sign of VRF. (b) Sagittal CBCT view showing a J-shaped radiolucency with total disruption of the buccal cortical plate of bone. (c) During microsurgical exploration, partial destruction of the buccal cortical plate of bone, total denudation of the buccal surface of the root, and a VRF on the midbuccal aspect of the root were evident after granulomatous tissue removal and root staining with methylene blue dye.

**Figure 3:**
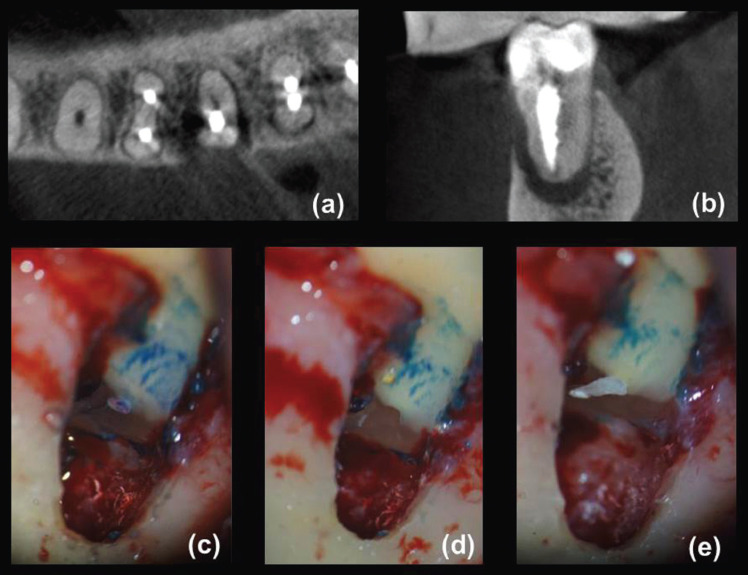
Mandibular left first molar endodontically treated with apical periodontitis accompanied by periodontal breakdown indicative of VRF. (a) Several fracture lines (solid arrows) and an accompanying periradicular bone defect with disruption of the buccal cortical plate can be observed on the axial CBCT scan. (b) Coronal CBCT scan showing buccal alveolar bone loss and J-shaped radiolucency (bone loss halo). (c) The surgical procedure revealed the presence of unprepared lateral ramifications of the root canal system in the apical third of the distal root (dashed arrows). (d) After root-end cavity preparation, no fracture lines were observed on resected root surface. (e) Root-end filling of the exposed canal was performed using EndoSequence root repair material.

**Figure 4:**
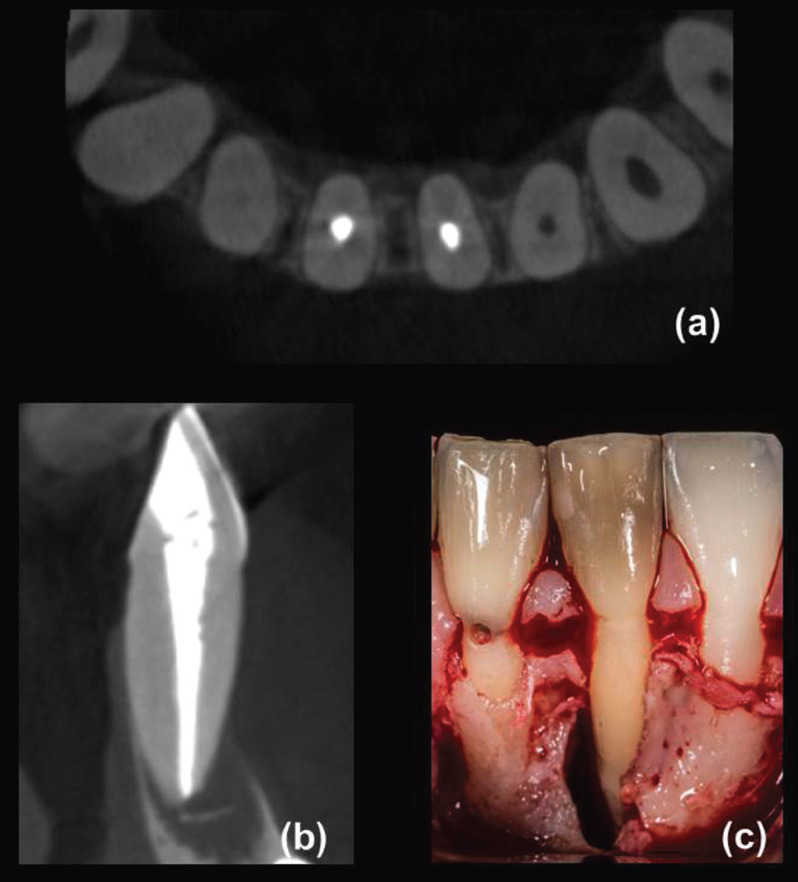
Mandibular left central incisor with persistent apical periodontitis and apicomarginal communication suggestive of VRF. (a) Axial CBCT view revealed the absence of fracture lines on the root structure. (b) Sagittal CBCT view showed a J-shaped defect extending from the periapical region to the alveolar crest. (c) Surgical approach showing a large bone defect due to apical pathosis with communication to the alveolar crest without evidence of fracture lines.

A total of 130 teeth in the same number of patients fulfilled the criteria for both CBCT and surgical assessment. Eighty participants were female (age range: 23-85 years, mean 56.5 years) and 50 were male (age range: 28-81 years, mean 53.0 years). In total, 65 teeth (18 incisors, 2 canines, 13 premolars and 32 molars) were diagnosed with VRFs after surgical exploration, including 56 teeth with visualized hypodense lines and apicomarginal bone loss (Fig. 1a-d) at CBCT examination and nine teeth with no detected hypodense lines on CBCT images (Fig. 2a-c), but with vertical bone loss. Of these, 64 teeth were extracted and one underwent root resection. Alternatively, a similar total of 65 teeth (12 incisors, 5 canines, 12 premolars and 36 molars) were confirmed as having symptomatic/asymptomatic apical periodontitis without VRF during surgical exploration. These latter, including 56 teeth with appearing hypodense lines and bone loss (Fig. 3a-e) and nine teeth with undetected hypodense lines and vertical bone loss (Fig. 4a-c), underwent EMS and followed up. As can be seen from Table 1, whereas no significant differences were identified among non-VRF and VRF groups with respect to the frequency of sinus tracts, tenderness to percussion, increased tooth mobility, and CBCT hypodense lines (all *P* >0.05, χ2 test), there was a significant greater proportion (*P* <0.05) of patients with tenderness to palpation, periodontal swelling/abscess, and probing depth ≥5 mm in the VRF group in comparison with that of non-VRF group. In addition, the evaluation of periapical status on CBCT images revealed significantly greater proportion of J-shaped hypodensities and smaller proportion of periapical hypodensities (all *P* <0.001, χ2 post hoc comparison test) in the VRF group in comparison with the non-VRF group.

**Table 1:**
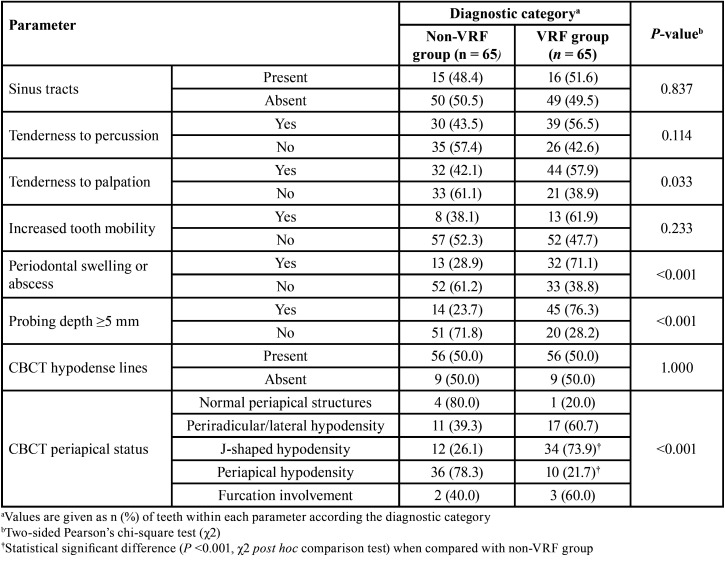
Summary of clinical and CBCT findings obtained from the study participants according diagnostic category.

-Predictive model for diagnosis of VRFs

The information presented in Table 2 describes the distribution of the grouped number of positive clinical and CBCT diagnostic criteria present in the patients and its relationship with the diagnosis of VRFs. As can be appreciated, those patients having more than three diagnostic criteria present had 8.80 times higher odds for the presentation of VRFs (95% CI, 2.89 – 26.79; *P* <0.001) when compared with those having only none or one. Moreover, after adjusting by age and gender, the predictive value of the model increased slightly. Conversely, those patients having two/three diagnostic criteria failed to reach a predictive value significantly higher than that obtained by the patients with non/one criteria for determining VRFs, even after adjusting age and gender.

**Table 2:**
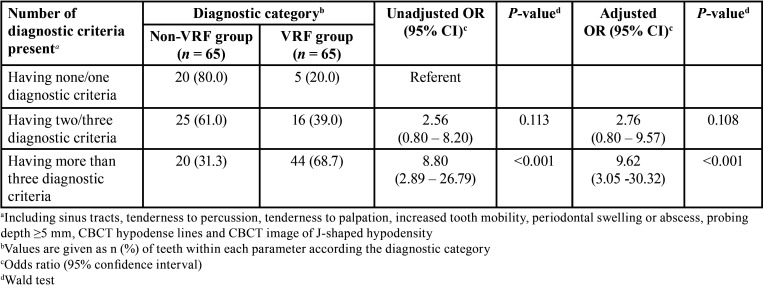
Predictive model for diagnosis of VRFs based on the number of positive clinical and CBCT diagnostic criteria present adjusted by age and gender.

-Bivariate comparisons of patient-, tooth-, and treatment-related predictor variables according to diagnostic categories of the study

 Table 3,  Table 4 and  Table 5 depict between-group comparisons of patient-, tooth-, or treatment-related predictor variables, respectively regarding to diagnostic categories. From Table 3 is evident that although no significant differences (*P* >0.05, χ2 test) between diagnostic categories regarding gender nor age stratum were detected, the proportion of patients with parafunctional habits was significantly greater (*P* <0.001) in the VRF group in comparison with that of the non-VRF group. Likewise, the only tooth-related predictor variable that contributed significantly (*P* <0.01) to VRFs, was the number of canals of the affected root (Table 4), whereas the type and quality of coronal restoration had a confounding effect on this association (*P* <0.20). It was also noteworthy that the effects of treatment-related predictor variables such as excessive root canal enlargement and absence intra-radicular posts were significantly related to the proportion of VRFs (Table 5, all *P* = 0.001), whereas the shaping and filling techniques also had a confounding influence on the results (*P* <0.20).

**Table 3:**
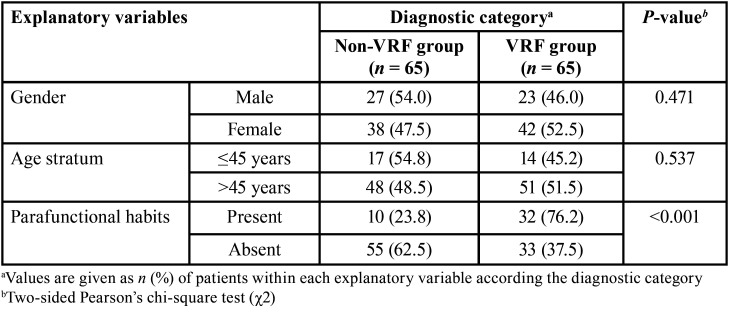
Between-group bivariate comparisons of patient-related predictor variables according the diagnostic category.

**Table 4:**
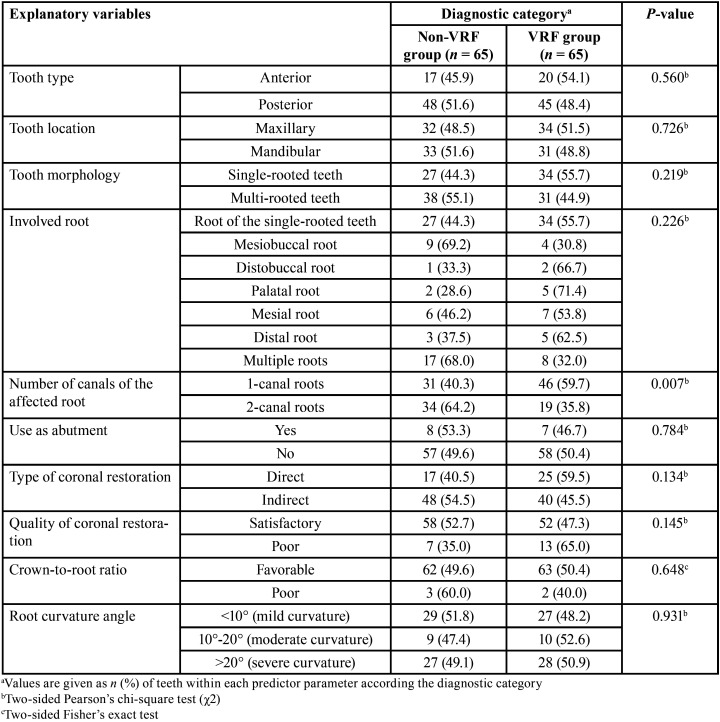
Between-group bivariate comparisons of tooth-related predictor variables according the diagnostic category.

**Table 5:**
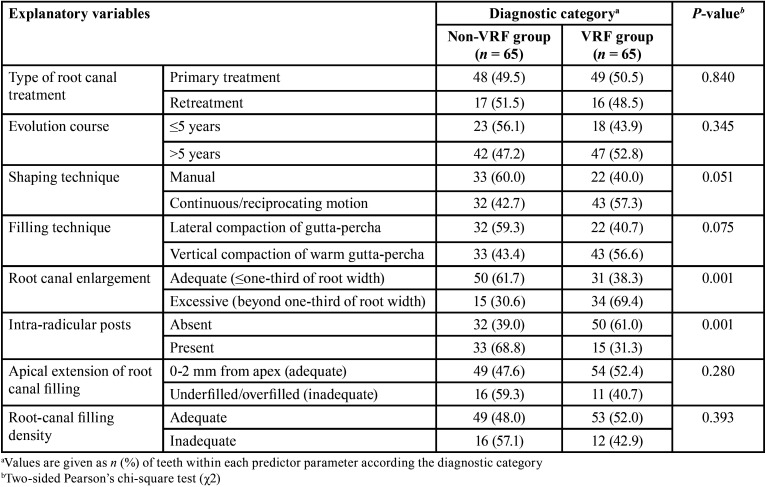
Summary of clinical and CBCT findings obtained from the study participants according diagnostic category.

-Analysis of univariate and multivariate binary logistic regression models for association with VRFs

Data resultant from univariate and multivariate binary logistic regression analyses of candidate variables for the association with VRFs before and after adjusting for the effects of confounders such as type of coronal restoration, quality of coronal restoration, shaping technique, and filling technique are summarized in Table 6. It was noTable that, the OR of VRFs was significantly increased (*P* <0.01, Wald’s test) for patients with parafunctional habits and in cases with one-canal roots, excessive root canal enlargement, and absence of intra-radicular posts. After adjusting individually for confounders selected from bivariate comparisons, all of these candidate predictor variables remained strongly and independently associated with VRFs (*P* <0.05). The Hosmer-Lemeshow goodness-of-fit test probability values ranged from 0.370 to 0.764, indicating that the logistic regression models were adequately adjusted. Also, the c-statistic values ranged from 0.669 to 0.677 in these adjusted models suggesting very good discrimination capacity.

**Table 6:**
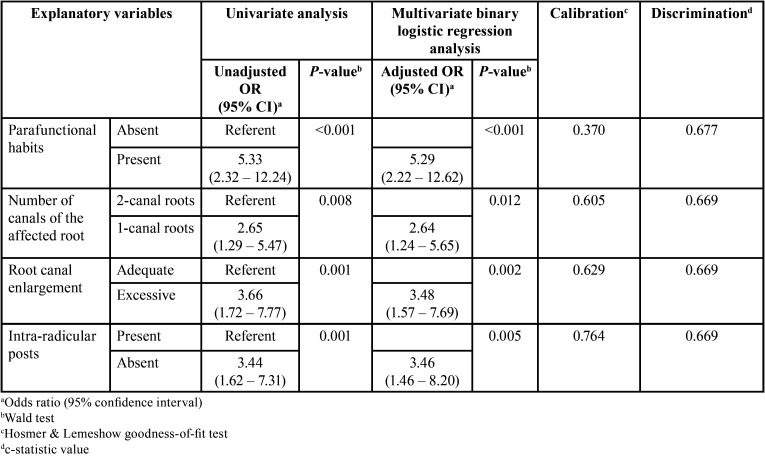
Initial and final models of multivariate binary logistic regression for association of significant predictor variables with VRFs after root canal treatment adjusting for non-significant confounding covariables.

## Discussion

It is widely acknowledged that detection of VRFs is an important diagnostic task in most clinical situations (2,16,19,26) since, whilst a false-negative result can lead to periodontal disease exacerbation and alveolar bone loss over time, a false-positive diagnosis might also result in unnecessary extraction of the tooth (26). Unfortunately, sometimes it is difficult to perform an accurate diagnosis due to the presence of non-specific signs/symptoms, especially in cases without evident separation of the adjacent segments (26,27) and variations in the presentation of VRFs (2,28). It is important to emphasize that this study was intended to detect *in vivo*, using EMS exploration, the presence of fracture lines in a representative sample of patients with suspected VRFs both clinically and radiographically, but including only cases in which the fracture could not be diagnosed at clinical examination in order to increase the internal validity of the final results.

Although CBCT has demonstrated important advantages over conventional intraoral radiographs for VRFs detection, its effectiveness cannot be assured (29). In this study the sensitivity and specificity values of CBCT imaging for diagnosis of VRFs were 86.2% and 13.8%, respectively, with PPV, NPV, and accuracy values of 50% each. While sensitivity was either higher or close similar than those which have been reported under clinical situations in earlier studies (2,30-32), the specificity, PPV, NPV, and accuracy were reduced markedly in comparison with those obtained by the same authors. The available evidence suggests that diagnostic performance of CBCT for VRF detection varies according to differences in CBCT scanning systems, including different voxel sizes, radiation doses, detector performances, and post-processing method (2,33), as well as dissimilarities due to the sample type (28), disease prevalence (34,35), and extent/width of the fracture (2,33,35). Based on the current data analyses, several methodological factors allow explaining the discrepancies. First, though this study confirmed with surgical exploration, the detection of VRFs through CBCT scans using the lowest possible voxel size (0.125 mm) with a limited of field vision as previously recommended (30), the sample was constituted exclusively by root-filled teeth. In this sense, previous studies have demonstrated that the specificity of CBCT may be reduced as a consequence of star-shaped streak artifacts and beam hardening associated with the presence of root fillings or metallic intracanal posts, which might mimic a fracture line (22,30,36-38). Second, 65 out of 130 teeth assessed proved to have VRFs yielding a prevalence rate estimated from sample of 50%. Taking into account that this result overcomes the prevalence in the overall population (9-11), a decrease in both specificity and predictive values can occur without systematic effect for sensitivity (34). Third, earlier *ex vivo* (1,33,38,39) and *in vivo* (2,28,31) studies have reported that the diagnostic performance of CBCT for the detection of VRFs depends on extent/width of the fracture, so that the thinner and limited the fracture, the more prone to misdiagnosis (1). In the current study, only cases with narrow VRFs without obvious separation of fractured fragments were included. This issue may have affected its detection rate on CBCT scans and therefore also the sensitivity of this test. Nonetheless, the detectability of VRFs by CBCT *in vitro* and *in vivo* does not depend only on the fracture widths, as detection accuracy among these two experimental methods varies widely (2,30). Whilst *in vitro* findings do not include patient factors, such as the effect of the surrounding tissues that could influence the quality of CBCT images (2,40) or the possibility of motion artifacts throughout scanning (36) that could hamper the detection of putative fracture plane (2), the decreased radiation dose related to *in vivo* scanning may contribute to poor image quality and reduced accuracy of the detection of fracture lines (41). Even so, it is recognized that *in vivo* studies would have been more realistic (37).

Taking in mind the results of this previous analysis, it should be necessary to point out that the use of CBCT in the diagnosis of VRFs should not be limited to the visualization of a fracture line (35). Rather, the cases must be assessed in combination with other clinical and radiographic signs and symptoms to eliminate false positive results. Accordingly, in the current study four specific diagnostic criteria were significantly more frequent in the VRF group, including tenderness to palpation, periodontal swelling/abscess, probing depth ≥5 mm, and radiological image of J-shaped hypodensity. Although these findings are in line with those obtained in earlier retrospective studies (15,19), it was noteworthy that only those cases having more than three diagnostic criteria present had a significant predictive value for determining VRFs. In consonance with the former, it has been stated that the more significant diagnostic factors are available, the greater the chance for the precision of VRF diagnosis (19).

Among the patient-related variables, only the presence of parafunctional habits was significantly associated with the percentage of VRFs in the univariate analysis and remained as a robust predictor when adjusted for covariables. While parafunctional habits have been described as predisposing factor for VRFs due the excessive, repetitive, and heavy masticatory stress applied to a tooth (42,43), some authors believe that this condition did not seem to be a significant factor (44). Reasons for disagreement might underlie in part in differences of the teeth strength and the intensity of bruxism/clenching (45). Regarding gender and age, the results have also been conflicting since while some studies appeals to one gender or another linking gender (16-17,44,46) and age differences (16,44,46), in concordance with the present results, other investigations did not report differences among gender (19) nor age stratum (17,19). These divergent findings may be due to variations in sample composition and experimental conditions.

Another broadly investigated issue, for which dissimilar results have been published, is the effect of the tooth-related variables on VRFs generation. Surprisingly in this study, the number of canals of the affected root was the foremost indicator, as those cases with one-canal roots were significantly associated with the percentage of VRFs in the univariate analysis and continued strongly and independently associated when adjusted for covariables. In contrast, although relatively little is known about the influence of the number of canals per root on the prevalence of VRFs, an *ex vivo* study performed in extracted mandibular molars (47) demonstrated that two-canal roots are much more prone to VRF than 1-canal roots. Taking into account that in this study, both single- and multi-rooted teeth were included, it would be possible to assume that those teeth with thin root walls and one-canal root, like observed in buccal roots of upper molars and lower incisors, may have accounted for such discrepancy.

An additional outstanding feature of the herein results was the strong and independent effect of two treatment-related variables in the proportion of VRFs. The covariables with significant impact were the excessive root canal enlargement and the absence of intra-radicular posts. Taking altogether, these treatment-related variables are important mainly because of the degree to which they may increase of stress of root canal walls (18). The present results parallel, at least in part, those reported by others who reported that altered distribution of the stress related to the enlargement of root canal diameter with further decrease of the wall dentine thickness decreases the root fracture strength (48). Additionally, consistent with the findings of previous studies (49,50), it seems that the presence of intra-radicular posts acts as a protective factor against VRFs. However, others have found significant associations between the presence of intra-radicular posts and VRFs (18,45), and other investigations have not confirmed a significant impact of this variable on VRFs (17,51). It is possible that the observed disagreements between the authors could be partially be explained by variability of intra-radicular posts analyzed (i.e., metal or fiber) and the cementation techniques used.

As a final point, two limitations were also apparent in the current study. First, the occlusal forces, occlusal contacts, postoperative root canal diameter, and peripheral root dentine thickness were not assessed. Since excessive occlusal forces and inadequate occlusal contacts, an unfavorable ratio between a large postoperative root canal diameter and small peripheral root dentine thickness may play an important role on VRFs generation. Further studies including precise functional analysis of these four variables are required. Second, the presence of beam hardening and streak artifacts could have compromised the quality of images. Hence, the use of methods for artifact reduction intended to enhance the contrast-to-noise ratio of the resulting CBCT images is encouraged.

## Conclusions

The findings of this study suggest that although the sensitivity of CBCT for VRFs detection is high, the risk of false-positive results related to its low specificity makes that all suspected cases must be confirmed by surgical exploration. VRFs cannot be reliably diagnosed by isolated clinical signs/symptoms; instead those teeth possessing more than three diagnostic criteria might be considered practically pathognomonic. The presence of parafunctional habits, one-canal roots, excessive root canal enlargement, as well as the absence of intra-radicular posts may act strongly and independently for the occurrence of VRFs in endodontically treated teeth.
